# Exploring the translational challenge for medical applications of ionising radiation and corresponding radiation protection research

**DOI:** 10.1186/s12967-022-03344-4

**Published:** 2022-03-18

**Authors:** Sophie Bockhold, Shane J. Foley, Louise A. Rainford, Riccardo Corridori, Annika Eberstein, Christoph Hoeschen, Mark W. Konijnenberg, Susan Molyneux-Hodgson, Graciano Paulo, Joana Santos, Jonathan P. McNulty

**Affiliations:** 1 Radiography and Diagnostic Imaging, School of Medicine, University College Dublin, Belfield, Dublin 4, Ireland; 2 COCIR, Brussels, Belgium; 3 Institute of Medical Engineering, Otto Von Guericke Universität Magdeburg, Magdeburg, Germany; 4 Department of Radiology and Nuclear Medicine, Erasmus Medical Centre, Rotterdam, Netherlands; 5 Nuclear Societies Research Group, University of Exeter, Exeter, UK; 6 Escola Superior de Tecnologia da Saúde, Instituto Politécnico de Coimbra, Coimbra, Portugal

**Keywords:** Translational medical research, Delphi study, Ionising radiation, Radiation protection

## Abstract

**Background:**

Medical applications of ionising radiation and associated radiation protection research often encounter long delays and inconsistent implementation when translated into clinical practice. A coordinated effort is needed to analyse the research needs for innovation transfer in radiation-based high-quality healthcare across Europe which can inform the development of an innovation transfer framework tailored for equitable implementation of radiation research at scale.

**Methods:**

Between March and September 2021 a Delphi methodology was employed to gain consensus on key translational challenges from a range of professional stakeholders. A total of three Delphi rounds were conducted using a series of electronic surveys comprised of open-ended and closed-type questions. The surveys were disseminated via the EURAMED Rocc-n-Roll consortium network and prominent medical societies in the field. Approximately 350 professionals were invited to participate. Participants’ level of agreement with each generated statement was captured using a 6-point Likert scale. Consensus was defined as median ≥ 4 with ≥ 60% of responses in the upper tertile of the scale. Additionally, the stability of responses across rounds was assessed.

**Results:**

In the first Delphi round a multidisciplinary panel of 20 generated 127 unique statements. The second and third Delphi rounds recruited a broader sample of 130 individuals to rate the extent to which they agreed with each statement as a key translational challenge. A total of 60 consensus statements resulted from the iterative Delphi process of which 55 demonstrated good stability. Ten statements were identified as high priority challenges with ≥ 80% of statement ratings either ‘Agree’ or ‘Strongly Agree’.

**Conclusion:**

A lack of interoperability between systems, insufficient resources, unsatisfactory education and training, and the need for greater public awareness surrounding the benefits, risks, and applications of ionising radiation were identified as principal translational challenges. These findings will help to inform a tailored innovation transfer framework for medical radiation research.

**Supplementary Information:**

The online version contains supplementary material available at 10.1186/s12967-022-03344-4.

## Background

Medicine has undergone rapid advancement in recent decades benefiting from the ongoing technological revolution and the dawn of personalised medicine, all made possible by a myriad of scientific discoveries [1]. Medical applications of ionising radiation and associated radiation protection research are a cornerstone of this medical evolution [2]. Exemplifying this flourishing progression in medical radiation research are the increasing number of novel imaging biomarkers [3], continuous expansion of interventional radiology applications [4], recent emergence of authorised theranostic radiopharmaceuticals [5–9], development of nanomedicine [10–12], establishment of new charged particle beam therapies [13, 14], and increasing utilisation of AI-based systems for image enhancement, segmentation, interpretation and object detection [15–17]. Moreover, our knowledge surrounding the adverse effects of human exposure to ionising radiation and the underlying biological pathways at play continue to expand and, in turn, radiation protection practices have become further enhanced [18–23]. Nevertheless, a longstanding issue is that clinical implementation continues to severely lag innovation and knowledge generation [1, 24]. Thus, a concerted effort is needed to develop robust translational roadmaps through which to overcome the hurdles encountered throughout the transition from research and development to wide-spread clinical implementation [1, 3].

There have been several translational challenges acknowledged for medical applications of ionising radiation over the years. These have included accounts of financial barriers [17, 24, 25], limited access and scarcity of resources [24, 26–28], cumbersome and ill aligned regulatory requirements [9, 28, 29], and insufficient data repositories [15, 17, 30]. The need for greater standardisation, communication, and collaboration regarding the conduct of medical radiation-based research at all levels has also been widely noted [24, 26, 28, 31–33]. Though, up to this point, reporting of translational challenges and proposed solutions to these challenges has been primarily ad hoc and project specific. To effectively translate ionising radiation research into wider clinical practice and ensure both the sustainability and competitiveness of medical radiation research at scale, a coordinated and integrated effort at the European level is needed. To this end, the objective of Work Package 5 within the larger EURAMED Rocc-n-Roll project was to analyse the research needs for innovation transfer in radiation based high-quality healthcare across Europe and develop an innovation transfer framework for medical ionising radiation research at scale. Specifically, Task 5.1 aimed to gain consensus on the key translational challenges causing this lack of innovation transfer and define a priority approach to addressing identified issues. The Delphi technique was employed to execute this task as it offers a validated means of gathering and synthesising expert opinion for the purposes of generating recommendations in medical research and has been used for similar studies addressing clinical research barriers, research priorities, and educational needs/core competencies across a range of healthcare disciplines, including emergency medicine, occupational therapy, and radiography [34–39].

## Methods

The study consisted of three Delphi rounds completed between March and September 2021. The first Delphi round began with a preliminary literature search to identify central aspects and commonly reported hurdles to clinical translation. Using prompts derived from the literature, an open-ended electronic survey was developed within SurveyMonkey® and distributed to all members of the Task 5.1 Working Group for their review and feedback prior to deployment. As a low-risk study, an exemption from full institutional review board approval was obtained from the UCD Human Research Ethics Committee – Life Sciences (Reference: LS-E-21–35-McNulty). Forty-six European leaders in medical radiation were then nominated by the Task 5.1 Working Group to participate in round one of the Delphi study for which respondents were given three weeks to generate a wide range of statements regarding key barriers to translation by way of the self-administered online survey. The survey link was distributed via email alongside a summary of the project’s aims and scope with participation being entirely voluntary and consent obtained within the survey form. Statements were submitted across four broad categories: Basic Research, Commercial Development, Clinical Implementation, and Education and Training. Submissions were subsequently consolidated, duplicates removed, and messaging refined by the core research team (authors SB, SF, and JM) through a series of online meetings to produce a final list of unique statements which were carried forward to the next round.

The second Delphi round engaged a broader panel of subject matter experts across all areas of medical radiation and radiation protection research – radiology, nuclear medicine, radiotherapy, and social science. An email invitation was sent to all members of the EURAMED Rocc-n-Roll Consortium in addition to the same 20 panellists who participated in round one of the Delphi process. Furthermore, eleven well-known international organisations were contacted by email asking for their support in distributing the survey link. Within the electronic survey tool, nominated individuals were asked to rate the extent to which they agreed (or disagreed) with each generated statement as a key translational challenge for radiation research via a 6-point Likert Scale (1 = Strongly Disagree to 6 = Strongly Agree). Statements which achieved consensus, defined as a median rating of ≥ 4 with ≥ 60% of responses in the upper tertile of the 6-point Likert Scale (i.e., Agree/Strongly agree), were automatically progressed forward to a third Delphi round. Concurrently, statements on the verge of consensus underwent a supplementary review process by the core research team with regard for both the literature and under-represented research areas for inclusion in the final iteration of the Delphi process. Respondents were also provided the opportunity to submit original statements at the end of the survey form and novel submissions progressed forward for expert rating.

Four weeks following the close of the second-round survey the same cross-disciplinary panel of experts was asked to rate the prioritised round two statements through a third iteration of the Delphi process to produce a final set of core translational challenges. Central tendency and dispersion were used to descriptively analyse aggregated data following each of the latter two Delphi rounds. The proportion of question responses in the upper tertile of the Likert Scale was also determined to identify and prioritise consensus statements. Moreover, a Wilcoxon Matched Pairs Signed Rank Test was conducted on each statement to assess the stability of panel responses across Delphi rounds. Descriptive and statistical analyses were conducted by a single member of the research team using Excel version 16.56 (Microsoft Corp., Redmond, USA) and SPSS version 27 (IBM Corp., New York, USA) respectively; statistical findings were subsequently reviewed by two additional members of the research team to increase validity of results.

## Results

### Panel composition

**Fig. 1:**
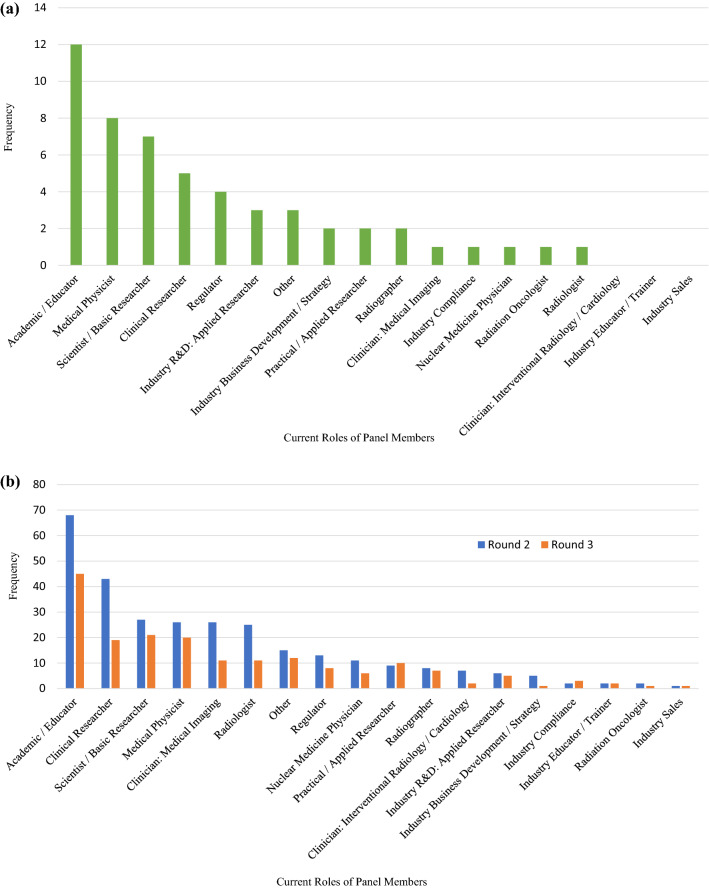
**a** Distribution of panelist roles in round one. **b** Comparison of the distribution of panelist roles between rounds two and three

From the forty-six individuals nominated to participate in round one, 20 individuals completed the open-ended survey, two declined to participate and the remaining 24 nominees were non-responders giving rise to a participation rate of 43%. Overall, there was good representation from the various sectors, with all but four respondents reporting they hold two or more roles within the fields of medical applications of ionising radiation and radiation protection research (Fig. 1a). The round two survey invitation reached approximately 350 professionals from which 130 individuals participated in statement ratings for a round two response rate of 37%. To facilitate an assessment of response stability, the third Delphi round called upon these same 130 panelists, though an attrition rate of 36% occurred between rounds. A comparison of the distribution of respondent roles across both rounds has been presented graphically in Fig. 1b. While all pre-identified roles were represented within the broader group, there was minimal participation from radiation oncologists despite efforts to recruit a balanced panel. Conversely, while each of the specific industry roles had minimal representation on their own, taken together a grand total of 12% (n = 16) of round two respondents were working within the industry sector, which was comparable to other represented disciplines. Overall, the distribution of roles remained somewhat similar across rounds; however, the proportion of respondents holding positions in clinical research, medical imaging, and radiology was notably higher in the preceding round, while basic research, medical physics, and practical/applied research were better represented in the latter round (Fig. 1b).

### Delphi process

**Fig. 2:**
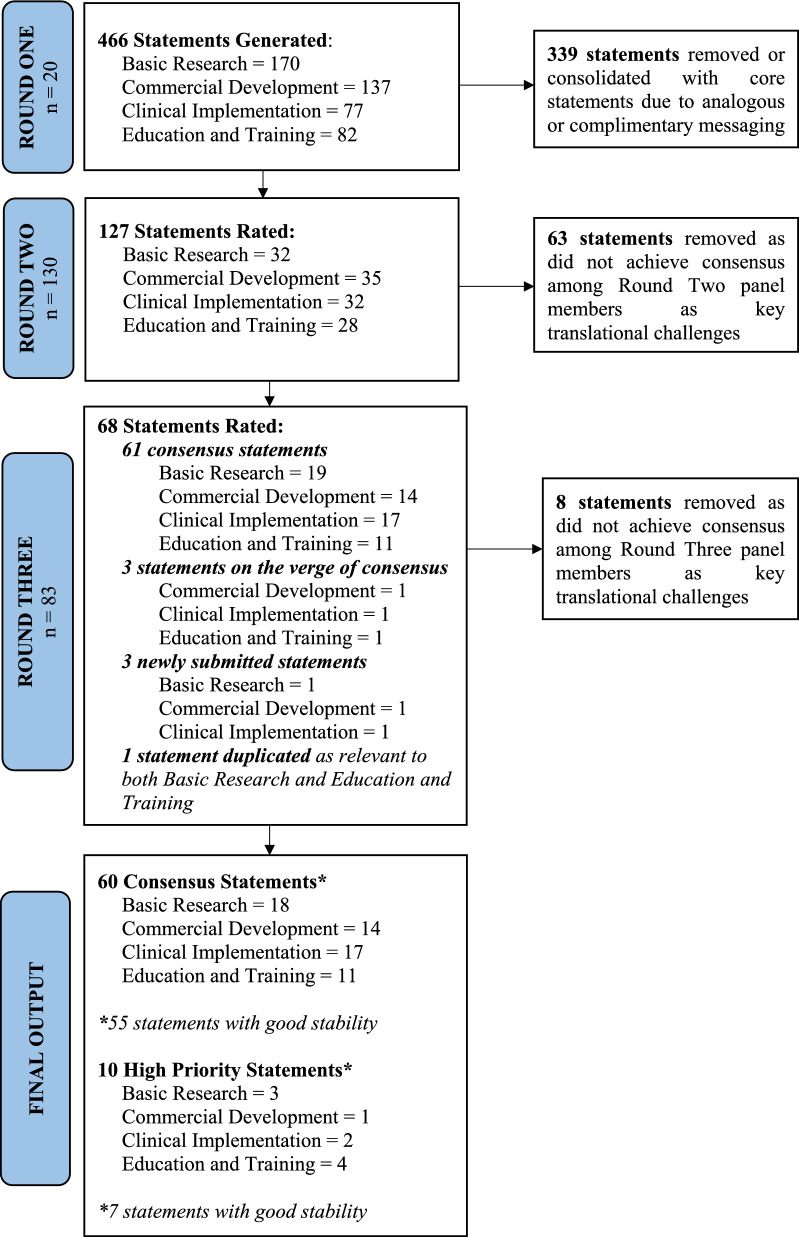
Flow diagram of the Delphi process

The first Delphi round produced a total of 466 statements. Upon removal of duplicate translational challenges and consolidation of statements with similar sentiments, 127 unique statements remained as per the following distribution: Basic Research 32, Commercial Development 35, Clinical Implementation 32, and Education and Training 28. When these statements were disseminated to the broader panel for rating, a total of 61 statements achieved the definition of consensus; as a result, these statements were automatically advanced for further rating in the third Delphi round. Moreover, three statements on the verge of consensus were progressed forward for their unique overarching topics and prominence throughout the literature, as well as three newly submitted statements advanced and one statement duplicated due to its relevance to both Basic Research and Education and Training categories. These additions resulted in a total of 68 statements carried forward to round three for a further iteration of the Delphi process. Subsequently, in response to panellist feedback which noted a disproportionate focus on diagnostic radiology, each of the 68 statements were further reviewed by project staff and statement wording was subtly modified to better encompass all pertinent disciplines where practicable and consensus achieved among staff members. A third and final Delphi round was then undertaken which identified a core set of 60 consensus statements. The overarching flow of statements through each of the three Delphi rounds is summarised by Fig. 2.

To define a priority approach for addressing the key challenges, consensus statements were then ranked first by median rating and then by the proportion of raters in agreement or strong agreement with each statement. Additionally, a Wilcoxon Matched Pairs Signed Rank Test revealed that the majority (n = 55) of consensus statements showed good stability across rounds. For a summary of all 60 ranked consensus statements by category alongside results of the stability analysis see Additional file 1: Table S1a–d). The list of 60 consensus statements was then further refined to highlight those challenges where ≥ 80% of respondents agreed or strongly agreed to narrow in further on the most pressing translational challenges to be addressed. Through this evaluation a high priority list of 10 hurdles to translation were identified (Table 1).

**Table 1:** Ranked high-priority consensus statements following three Delphi rounds and results of stability analysis

Category	Ranking	Statement	Round 2			Round 3			Wilcoxon Signed Rank Test	
			Median Rating	IQR	Percent (%) in Top Tertile	Median Rating	IQR	Percent (%) in Top Tertile	Z Score	P-value
Basic Research	1	Commercial software is often a black box. When using clinical data (e.g., images) in basic research it is difficult to judge what happened to the data (e.g., post-processing effects), which can lead to biased study results	5	2	71.43	5	1	84.21	**−2.475**	0.013*
Basic Research	2	Robust and efficient database structures that facilitate research across different repositories/platforms through secure data storage and information exchange are needed	5	1	88.6	5	1	83.54	**−**0.29	0.772
Clinical Implementation	3	The translation of novel research not only requires personnel (e.g., specialist clinical staff across multiple professions) but also access to high-end, or state of the art, imaging and/or radiotherapy equipment. Such conditions are heterogeneous in Europe, i.e., some research will only be conducted at very few institutes or with very few healthcare providers	5	2	74.07	5	1	83.54	**−**0.657	0.511
Education & Training	4	Experience and background knowledge varies greatly	5	2	74.34	5	0	83.12	**−**1.418	0.156
Education & Training	5	Adequate training is often a challenge as clinical demands minimise the number of staff and average time spent on end user training (often working around clinical work/examinations/procedures)	5	1	71.05	5	1	81.82	**−**0.526	0.599
Clinical Implementation	6	The clinical setting is usually very complex with multiple technologies, and software systems, working together; correct integration and connections are crucial but often difficult	5	1	62.5	5	0	80.77	**−2.316**	0.021*
Education & Training	7	There is a need for multidisciplinary approaches to education and training that incorporate a team of educators with radiation protection expertise from a range of professions/disciplines	5	1	83.93	5	1	80.52	**−1.975**	0.048*
Basic Research	8	There is a lack of funding, as well as a lack of funding opportunities, particularly for basic radiation protection research	5	1	75.93	5	1	80.26	**−**0.364	0.716
Education & Training	9	General awareness (by the public and other healthcare workers) of the benefits, risks, and applications of ionising radiation needs improvement	5	1	82.35	5	1	80.25	**−**0.64	0.522
Commercial Development	10	Access to modern technology/up-to-date equipment in radiology, nuclear medicine, or radiotherapy is limited by financial factors due to the high cost of resources, with end-users often lagging behind commercial development	5	1	62	5	1	80	**−**1.675	0.094

^*^Statistically significant result indicating lack of stability across Delphi rounds

## Discussion

The laborious and often unsuccessful transfer of medical innovations into clinical practice has been an issue at the forefront of medical research for decades and the focus of much infrastructural and strategic reform at the national and international levels since the turn of the century [1, 25, 40–42]. The clinical and translational research continuum is intensively promoted as the gold standard through which to actualise the untapped potential of scientific discoveries [1, 43]; however, the core roadmap must be further adapted to best meet product and application specific needs with no one size fits all formula for innovation transfer [19, 40]. The extensive list of translational challenges identified through the presented Delphi work solidifies the need for an adapted innovation transfer framework specific to clinical applications of ionising radiation.

Through the Delphi process a distinct set of sixty translational challenges was identified from which ten high priority issues emerged which require immediate attention (Table 1). A prominent theme amongst the top ranked translational challenges was a lack of interoperability and information exchange. The statement which achieved the greatest level of combined agreement and stability across Delphi rounds being “robust and efficient database structures that facilitate research across different repositories/platforms through secure data storage and information exchange are needed.” This consensus statement is well aligned with the 2017 Common Strategic Research Agenda (SRA) for medical radiation protection, though not one of the agenda’s primary research topics, wherein a problematic degree of technological variability was acknowledged and an interdisciplinary collaboration for the development of harmonised procedures and standards of practice proposed as a potential solution to this problem [31]. Structured reporting and standardised coding systems were also promoted within the SRA and have been reported throughout the broader literature as a necessary means to facilitate information transfer [2, 31]. Similarly, limitations brought about by vendor-specific technology, heterogeneous data, and lack of data security are at the core of the NIH National Center for Data to Health’s (CD2H) research strategy [44]. The European Society for Translational Medicine (EUSTM) has also emphasised the importance of a robust data management framework built upon the principles of data integration, regulatory compliance, security, and scalability for successful translation of medical research [45]. The current Delphi work’s identification of “[complex clinical settings] with multiple technologies, and software systems working together” provides further support for the promotion of good data management systems and standardised coding, while the statement “Commercial software is often a black box” highlights the need for close collaboration between clinical research centres and industry when developing software and database structures. However, the latter two consensus statements lacked stability across Delphi rounds indicating these issues may not be as pressing as the need for robust and efficient database structures.

Financial constraints was another common theme that arose out of the Delphi work, with approximately 80% (n = 52 and n = 61, respectively) of statement raters having agreed or strongly agreed with the following two statements in round three: “access to modern technology/up-to-date equipment in radiology, nuclear medicine, or radiotherapy is limited by financial factors due to the high cost of resources, with end-users often lagging behind commercial development” and “there is a lack of funding, as well as a lack of funding opportunities, particularly for basic radiation protection research.” These findings are not entirely unexpected given insufficient funding has been a commonly cited barrier to translation for both the medical radiation and wider medical research community [26–28, 43]. Though the continued prominence of this issue contradicts the influx of funding for translational research projects in recent decades, indicating a re-evaluation of current funding distribution may be needed [25, 46]. Insufficient access to personnel and equipment was also identified as a key translational challenge. A finding that converges with a recent study out of the United Kingdom that identified a general lack of resources (funding, staffing, and infrastructure) as one of four primary contributors to the inefficient set-up of radiotherapy trials [27]; though these findings may be due in part to the repercussions of the United Kingdom’s recent exit from the European Union [47–49]. Looking further into the staffing shortage, a survey of radiotherapy research staff revealed that most clinical centres had ≤ 1 whole time equivalent physicist, research nurse, data manager, and radiographer working within their radiotherapy research centre [33]. The existence of a severe staffing shortage further supported by the European Association of Nuclear Medicine (EANM) Internal Dosimetry Task Force’s 2015 survey which found that only 68% of radionuclide therapies involved a medical physicist [50]. Taken together with the high priority challenges identified through the current Delphi study and the alarming radiology workforce shortages reported across Europe, these survey findings shed light on a severe drought in the current medical radiation workforce which must be addressed if the field of radiation research is to realise the tremendous potential of its scientific discoveries [25, 51–54].

One proposed solution to the current workforce shortage is to increase the number of professionals trained in clinical and translational research. This solution echoes the prominence of education and training within the strategic agenda of medical societies and research funding bodies across North America and Europe [25, 31, 40]. However, the findings from the current study demonstrate the need for a more standardised and multidisciplinary approach to education and training. Two of the top ten translational challenges identified stating: “Experience and background knowledge varies greatly” and “there is a need for multidisciplinary approaches to education and training that incorporate a team of educators with radiation protection expertise from a range of professions/disciplines.” It must also be stated that training programmes cannot solely be directed at young professionals. Consensus around “adequate training often [being] a challenge as clinical demands minimise the number of staff and average time spent on end user training (often working around clinical work/examinations/procedures)” signifies that greater emphasis must also be placed on continuing professional development. Protected clinician/researcher time should be dedicated for both teaching & learning, particularly if staff are to stay up to date with the rapid advancements to technology and techniques. “General awareness (by the public and other healthcare workers) of the benefits, risks, and applications of ionising radiation [also] needs improvement.” This consensus statement converges with the trend towards patient-centric approaches and shared decision medicine [41]; though community access to both research data and scientific literature must be improved, and efforts directed at ensuring research outcomes are communicated in a manner easily understood by the general public. Most importantly, further work is needed to develop an innovation transfer framework that engages patients as key stakeholders [41].

The systematic and structured Delphi technique has enabled consensus on which translational challenges are most affecting the radiation research community today. Nevertheless, there are several limitations to the current study that must be noted, not least of which include the study’s self-selection sampling method and self-administered survey design. Additionally, consolidation and refinement of developed statements was conducted via content analysis, hence a degree of interpretation was required. The imbalanced panel composition and minimal participation from radiation oncologists also represents a potential limitation of the current findings; the translational challenges identified via the study panels being potentially not as relevant to the field of radiotherapy compared to radiology and nuclear medicine applications. Nonetheless, the Delphi work presented herein provides valuable insight into the current roadblocks which prevent medical radiation applications and protection research from achieving wide-spread clinical use.

## Conclusion

A lack of interoperability to facilitate information exchange, insufficient resources, unsatisfactory education and training, and the need for greater public awareness around the benefits, risks and applications of ionising radiation were identified as central issues in need of urgent attention. While these translational barriers are well-aligned with previous reports throughout the literature, the structured Delphi process provides added value to the existing body of knowledge. As a next step, presented consensus statements will be used to inform the development of a bespoke innovation transfer framework for medical applications of ionising radiation and corresponding radiation protection research. The resulting framework will provide a tool to help overcome key translational challenges currently facing the European radiation research community and help to inform future research and development work in medical applications of ionising radiation for maximum benefit to patients, professionals, and the wider European and global community.

## Supplementary Information

**Additional file 1: Table S1a.** Basic Research—Ranked consensus statements following three Delphi rounds and results of stability analysis.** b**. Commercial Development—Ranked consensus statements following three Delphi rounds and results of stability analysis.** c**. Clinical Implementation—Ranked consensus statements following three Delphi rounds and results of stability analysis.** d**. Education and Training—Ranked consensus statements following three Delphi rounds and results of stability analysis

## Data Availability

The datasets used and/or analysed during the current study are available from the corresponding author on reasonable request.
