# Supplementary material for: Exploring the translational challenge for medical applications of ionising radiation and corresponding radiation protection research
Source: J Transl Med. 2022 Mar 18;20:137. doi: 10.1186/s12967-022-03344-4 (PMC8932076; doi:10.1186/s12967-022-03344-4)
Supplement: Supplementary file 1 — Additional file 1: Table S1a. Basic Research—Ranked consensus statements following three Delphi rounds and results of stability analysis. b. Commercial Development—Ranked consensus statements following three Delphi rounds and results of stability analysis. c. Clinical Implementation—Ranked consensus statements following three Delphi rounds and results of stability analysis. d. Education and Training—Ranked consensus statements following three Delphi rounds and results of stability analysis [file 12967_2022_3344_MOESM1_ESM.docx]

## Supplementary Material

**Supplementary Table 1a.** Basic Research: ranked consensus statements following three Delphi rounds and results of stability analysis.

| **Category: Basic Research** | | **Round 2** | | | **Round 3** | | | **Wilcoxon Signed Rank Test** | |
| --- | --- | --- | --- | --- | --- | --- | --- | --- | --- |
| **Ranking** | **Statement** | **Median Rating** | **IQR** | **Percent (%) in Top Tertile** | **Median Rating** | **IQR** | **Percent (%) in Top Tertile** | **Z Score** | **P-value** |
| 1 | Commercial software is often a black box. When using clinical data (e.g., images) in basic research it is difficult to judge what happened to the data (e.g., post-processing effects), which can lead to biased study results. | 5.00 | 2.00 | 71.43 | 5.00 | 1.00 | 84.21 | **-2.475** | **0.013^a^** |
| 2 | Robust and efficient database structures that facilitate research across different repositories/platforms through secure data storage and information exchange are needed. | 5.00 | 1.00 | 88.60 | 5.00 | 1.00 | 83.54 | -0.290 | 0.772 |
| 3 | There is a lack of funding, as well as a lack of funding opportunities, particularly for basic radiation protection research. | 5.00 | 1.00 | 75.93 | 5.00 | 1.00 | 80.26 | -0.364 | 0.716 |
| 4 | The lengthy approval process is a challenge, particularly for small companies / start-ups with limited funding and resources. | 5.00 | 2.00 | 70.21 | 5.00 | 0.00 | 78.79 | -0.998 | 0.318 |
| 5 | There is a lack of European dose-imaging data repositories. | 5.00 | 1.00 | 73.64 | 5.00 | 2.00 | 73.68 | -0.318 | 0.751 |
| 6 | The ability to handle very large and complex data sets (i.e., suitable computing power and use of artificial intelligence) is a challenge in medical radiation research (i.e., radiology, nuclear medicine, radiotherapy). | 5.00 | 1.00 | 73.04 | 5.00 | 1.00 | 73.42 | -0.435 | 0.663 |
| 7 | Prototyping / product testing infrastructures are often expensive and difficult to finance, particularly in the academic setting. | 5.00 | 1.00 | 79.61 | 5.00 | 2.00 | 72.06 | -1.374 | 0.169 |
| 8 | The EU laws around funding are complex and it is challenging to keep abreast of what grant/funding opportunities are available and when. | 5.00 | 1.00 | 73.79 | 5.00 | 2.00 | 71.23 | -0.551 | 0.581 |
| 9 | It is difficult to secure investors without giving up intellectual property. | 5.00 | 1.00 | 63.22 | 5.00 | 1.00 | 70.97 | -1.554 | 0.120 |
| 10 | Need for more partnerships between the public and private sectors (i.e., research institutions/academia and commercial developers) to allow new technology, devices, methodologies, therapies, and radiopharmaceuticals the opportunity to break into the market. | 5.00 | 1.00 | 73.33 | 5.00 | 1.00 | 70.27 | -0.878 | 0.380 |
| 11 | Challenge to keep up to date with servicing and software updates for medical radiation equipment and technology across all disciplines (i.e., radiology, nuclear medicine, radiotherapy). | 5.00 | 1.00 | 67.62 | 5.00 | 1.00 | 69.74 | -1.240 | 0.215 |
| 12 | There is a gap for medium sized financing needs (i.e., either a very small amount of money or really big investments are possible, but in between is difficult). | 5.00 | 1.00 | 60.00 | 5.00 | 1.00 | 69.35 | -1.061 | 0.289 |
| 13 | Variation in software systems, procedure coding, acquisition protocols, and RIS/PACS interoperability makes clean big data difficult to acquire. | 5.00 | 2.00 | 72.73 | 5.00 | 1.25 | 67.50 | -0.401 | 0.689 |
| 14 | Lack of transparency regarding the algorithm used for dose calculations in different software. | 5.00 | 1.25 | 71.43 | 5.00 | 1.00 | 67.11 | -1.221 | 0.222 |
| 15 | Need for a harmonised approach to translational data sharing, which incorporates standardised data formatting / data coding, is supported by legislation, and is respectful of data privacy. | 5.00 | 0.00 | 78.38 | 5.00 | 1.00 | 66.67 | **-1.980** | **0.048^a^** |
| 16 | Quality assurance for IT systems is a challenge. | 5.00 | 1.00 | 69.64 | 5.00 | 1.00 | 66.67 | -0.042 | 0.967 |
| 17 | There is often a lack of knowledge around intellectual property (IP) / patenting in the research setting. | 5.00 | 1.00 | 63.46 | 5.00 | 1.00 | 63.24 | -0.126 | 0.900 |
| 18 | High costs associated with software and IT solutions present a barrier to implementation of suitable systems. | 5.00 | 1.00 | 60.75 | 5.00 | 1.00 | 62.16 | -1.679 | 0.093 |

^a^Statistically significant results, which indicate a lack of stability in panellists’ responses across Delphi rounds.

**Supplementary Table 1b.** Commercial Development: Ranked consensus statements following three Delphi rounds and results of stability analysis.

| **Category: Commercial Development** | | **Round 2** | | | **Round 3** | | | **Wilcoxon Signed Rank Test** | |
| --- | --- | --- | --- | --- | --- | --- | --- | --- | --- |
| **Ranking** | **Statement** | **Median Rating** | **IQR** | **Percent (%) in Top Terile** | **Median Rating** | **IQR** | **Percent (%) in Top Terile** | **Z Score** | **P-value** |
| 1 | Access to modern technology / up-to-date equipment in radiology, nuclear medicine, or radiotherapy is limited by financial factors due to the high cost of resources, with end-users often lagging behind commercial development. | 5.00 | 1.00 | 62.00 | 5.00 | 1.00 | 80.00 | -1.675 | 0.094 |
| 2 | The lack of harmonisation surrounding implementation of EU Regulations/Directives across member states, in particular the Basic Safety Standards Directive (BSSD) and General Data Protection Regulations (GDPR), in addition to the variable regulations across different countries and regions of the world presents translational challenges. | 5.00 | 1.00 | 61.22 | 5.00 | 0.00 | 78.87 | **-2.408** | **0.016^a^** |
| 3 | Quality Assurance and Quality Control, with respect to radiation protection principles (justification, optimisation) and other regulatory requirements, need to be better foreseen during the development of novel techniques / technologies / therapies and developers must have regard for the accessibility of necessary equipment and/or software required by the end user to perform QA/QC testing. | 5.00 | 1.00 | 73.96 | 5.00 | 0.00 | 77.78 | -1.141 | 0.254 |
| 4 | Translating IP into clinical practice often involves significant investment (financial and time) due to long evaluation processes, short patent lifetimes, quick technology development, and difficulties proving newly developed software is patentable, which presents a barrier to investment and EU/Regional competitiveness. | 5.00 | 1.00 | 68.97 | 5.00 | 1.00 | 73.33 | -0.564 | 0.573 |
| 5 | There is a lack of accessible (patient) data repositories. | 5.00 | 2.00 | 72.64 | 5.00 | 1.00 | 72.73 | -0.112 | 0.911 |
| 6 | There is a lack of specific funding for the commercialisation of radiation protection research. | 5.00 | 1.00 | 66.67 | 5.00 | 1.00 | 71.01 | -0.361 | 0.718 |
| 7 | Navigating EU legislations and CE-marking of medical devices is an arduous and (very) costly process, which presents an obstacle for the development of new technologies, particularly for small companies/start-ups. | 5.00 | 1.00 | 72.09 | 5.00 | 1.00 | 70.15 | -0.201 | 0.840 |
| 8 | Implementation of software solutions is difficult as it often requires a great deal of effort/resources, change management, and presents a risk to the organisation implementing the software (e.g., data loss, negative impact on other systems, etc.). | 5.00 | 1.00 | 61.39 | 5.00 | 1.00 | 68.92 | -0.158 | 0.875 |
| 9 | There is a lack of knowledge and experience with regards to market authorisation and CE marking; moreover, it is difficult to find and access experienced specialists in the field (e.g., adequate Quality Assurance Regulatory Assurance personnel). | 5.00 | 1.00 | 65.48 | 5.00 | 1.00 | 68.66 | -0.793 | 0.428 |
| 10 | The question of patent/IP ownership can be challenging in projects where an industry partner collaborates with healthcare and academia. | 5.00 | 1.00 | 62.77 | 5.00 | 1.00 | 67.69 | -0.476 | 0.634 |
| 11 | There is a shortage of IT specialists competent in radiation research. | 5.00 | 1.00 | 68.57 | 5.00 | 1.00 | 64.38 | -1.608 | 0.108 |
| 12 | Market authorisation often requires partial authorisations provided by different administrations with a need for better integration of the various regulations/regulatory processes (e.g., Euratom directive not well integrated with market authorisation processes and regulations such as EU MDR). | 5.00 | 1.00 | 60.00 | 5.00 | 1.00 | 63.79 | -0.478 | 0.632 |
| 13 | Regulatory matters, in particular compliance with GDPR and its various interpretations in different countries, presents a challenge for developing and implementing novel IT systems / software and makes it difficult for industry to collaborate with healthcare. | 5.00 | 1.00 | 59.00 | 5.00 | 1.00 | 61.97 | -0.132 | 0.895 |
| 14 | Regulatory bodies have struggled to keep up with the rapid transformation and growth of the healthcare sector. This is exemplified by the lack of notifying bodies within the medical device industry. | 5.00 | 1.00 | 61.36 | 5.00 | 1.00 | 61.29 | -0.444 | 0.657 |

^a^Statistically significant results, which indicate a lack of stability in panellists’ responses across Delphi rounds.

**Supplementary Table 1c.** Clinical Implementation: Ranked consensus statements following three Delphi rounds and results of stability analysis.

| **Category: Clinical Implementation** | | **Round 2** | | | **Round 3** | | | **Wilcoxon Signed Rank Test** | |
| --- | --- | --- | --- | --- | --- | --- | --- | --- | --- |
| **Ranking** | **Statement** | **Median Rating** | **IQR** | **Percentage (%) in Top Tertile** | **Median Rating** | **IQR** | **Percentage (%) in Top Tertile** | **Z Score** | **P-value** |
| 1 | The translation of novel research not only requires personnel (e.g., specialist clinical staff across multiple professions) but also access to high-end, or state of the art, imaging and / or radiotherapy equipment. Such conditions are heterogeneous in Europe, i.e., some research will only be conducted at very few institutes or with very few healthcare providers. | 5.00 | 2.00 | 74.07 | 5.00 | 1.00 | 83.54 | -0.657 | 0.511 |
| 2 | The clinical setting is usually very complex with multiple technologies, and software systems, working together; correct integration and connections are crucial but often difficult. | 5.00 | 1.00 | 62.50 | 5.00 | 0.00 | 80.77 | **-2.316** | **0.021^a^** |
| 3 | Consensus is needed on required image quality and how to quantify image quality in order for standard procedures to be implemented; both are currently missing. | 5.00 | 1.00 | 71.43 | 5.00 | 1.00 | 79.49 | -1.643 | 0.100 |
| 4 | QA is a big challenge for AI based applications, especially with respect to meaningful testing and understanding / evaluating limitations. | 5.00 | 1.00 | 84.26 | 5.00 | 1.00 | 79.22 | -1.074 | 0.283 |
| 5 | Dosimetric information (in radiology, nuclear medicine, radiotherapy) acquisition protocol details, and images are stored digitally, but are not easily shared between institutes; this creates a notable lack of communication / knowledge sharing, which hinders good clinical practise. | 5.00 | 1.00 | 78.38 | 5.00 | 1.00 | 75.64 | -0.108 | 0.914 |
| 6 | European standards and requirements are often formally adopted, but not well implemented in national and local practises. For example, the lack of harmonised standards for implementation of the EU Medical Device Regulation (MDR) and clinical trials regulations lead to variable interpretations of clinical evaluation requirements. | 5.00 | 1.00 | 63.11 | 5.00 | 1.00 | 75.34 | -1.645 | 0.100 |
| 7 | There is often a lack of funding to conduct pilot studies / early phase clinical trials. | 5.00 | 1.00 | 66.04 | 5.00 | 2.00 | 73.33 | -0.525 | 0.600 |
| 8 | Systems need country (and sometimes even region) specific set-up to fulfill the regulatory obligations, which can be cumbersome. | 5.00 | 1.00 | 61.68 | 5.00 | 1.00 | 72.86 | -0.931 | 0.352 |
| 9 | New technologies / therapies are not easily adopted by insurance companies. | 5.00 | 2.00 | 69.89 | 5.00 | 2.00 | 71.43 | -0.239 | 0.811 |
| 10 | Financing is oriented at suspected market shares, which presents an obstacle (e.g., for the development of a new radionuclide compound). | 5.00 | 1.00 | 53.33 | 5.00 | 1.00 | 70.18 | -0.177 | 0.076 |
| 11 | The lack of harmonisation hinders broad clinical implementation and makes comparison of new and existing methods more complex. | 5.00 | 1.00 | 70.37 | 5.00 | 1.00 | 69.33 | -0.621 | 0.534 |
| 12 | There are limited funding opportunities for clinical implementation. | 5.00 | 1.00 | 69.23 | 5.00 | 1.00 | 68.06 | -0.330 | 0.741 |
| 13 | Guidelines, recommendations, and clinical practises are often lagging behind modern technology / techniques / therapies. | 5.00 | 1.00 | 67.86 | 5.00 | 1.00 | 66.67 | -0.365 | 0.715 |
| 14 | Dose protocols (in radiology, nuclear medicine, radiotherapy) are often tailored to local subjective preferences as opposed to being evidence based; greater harmonisation of evidence-based dosimetry protocols is needed. | 5.00 | 1.25 | 63.89 | 5.00 | 2.00 | 65.38 | -1.481 | 0.139 |
| 15 | Pilot projects commonly require healthcare specialists for successful deployment, which may be a problem to secure (i.e., taking radiologists from clinical duties to pilot projects / lack of interest and motivation from clinicians). | 5.00 | 1.00 | 64.49 | 5.00 | 1.00 | 64.94 | -0.124 | 0.901 |
| 16 | The long authorisation process, due to various regulations that need to be followed (i.e., need for approval / clearance from national agencies in addition to ethical approvals), is a hurdle to initiating pilot studies / early phase clinical trials. | 5.00 | 1.00 | 66.67 | 5.00 | 1.00 | 64.86 | -0.258 | 0.797 |
| 17 | Standardisation of medical behaviour needs more than guidelines; it requires convincement, incentives and sanctioning. | 5.00 | 1.00 | 63.21 | 5.00 | 1.00 | 64.00 | -0.715 | 0.474 |

^a^Statistically significant results, which indicate a lack of stability in panellists’ responses across Delphi rounds.

**Supplementary Table 1d.** Education and Training: Ranked consensus statements following three Delphi rounds and results of stability analysis.

| **Category: Education & Training** | | **Round 2** | | | **Round 3** | | | **Wilcoxon Signed Rank Test** | |
| --- | --- | --- | --- | --- | --- | --- | --- | --- | --- |
| **Ranking** | **Statement** | **Median Rating** | **IQR** | **Percent (%) in Top Tertile** | **Median Rating** | **IQR** | **Percent (%) in Top Tertile** | **Z Score** | **P-value** |
| 1 | Experience and background knowledge varies greatly. | 5.00 | 2.00 | 74.34 | 5.00 | 0.00 | 83.12 | -1.418 | 0.156 |
| 2 | Adequate training is often a challenge as clinical demands minimise the number of staff and average time spent on end user training (often working around clinical work / examinations / procedures). | 5.00 | 1.00 | 71.05 | 5.00 | 1.00 | 81.82 | -0.526 | 0.599 |
| 3 | There is a need for multidisciplinary approaches to education and training that incorporate a team of educators with radiation protection expertise from a range of professions/disciplines. | 5.00 | 1.00 | 83.93 | 5.00 | 1.00 | 80.52 | **-1.975** | **0.048^a^** |
| 4 | General awareness (by the public and other healthcare workers) of the benefits, risks, and applications of ionising radiation needs improvement. | 5.00 | 1.00 | 82.35 | 5.00 | 1.00 | 80.25 | -0.640 | 0.522 |
| 5 | Clinical translation with regard to education and training in radiation safety requires commitment from clinicians and an understanding of the risks associated with ionising radiation, which is not always the case outside of radiology. | 5.00 | 2.00 | 71.05 | 5.00 | 0.25 | 78.75 | -0.688 | 0.491 |
| 6 | The use of installed technology is not fully maximised (and opportunities and pitfalls not fully understood) due to a lack of education and training and insufficient educational resources. | 5.00 | 1.00 | 66.37 | 5.00 | 1.00 | 78.21 | -1.574 | 0.116 |
| 7 | There is an unmet need for recurrent / continuous training of end users, particularly in the case of new staff who did not participate in the initial training session(s) provided by the manufacturer upon installation. | 5.00 | 0.50 | 74.77 | 5.00 | 0.00 | 77.92 | -0.596 | 0.551 |
| 8 | There is a lack of dedicated education, and continuing professional development (CPD), time for health professionals to implement consistent, up to date, evidence-based practises. | 5.00 | 1.00 | 59.26 | 5.00 | 0.00 | 75.32 | -1.008 | 0.313 |
| 9 | There is a lack of harmonisation with regard to education and training in radiation protection across Europe, which allows for non-harmonised certification procedures, variable education levels/degrees, and subjectivity in the certification of experts/specialists. | 5.00 | 1.00 | 72.07 | 5.00 | 1.25 | 71.05 | -0.156 | 0.876 |
| 10 | Hospital managers are often not aware of the importance for health professionals to develop key selection criteria (KSC) in Radiation Protection. | 5.00 | 2.00 | 68.69 | 5.00 | 2.00 | 70.83 | -1.208 | 0.227 |
| 11 | Lack of high-quality training resources and a need for more resources to be made available online for maximum impact. | 5.00 | 1.00 | 66.07 | 5.00 | 1.00 | 69.23 | -1.020 | 0.308 |

^a^Statistically significant results, which indicate a lack of stability in panellists’ responses across Delphi rounds.
